# Multiscale micromechanics modeling of plant fibers: upscaling of stiffness and elastic limits from cellulose nanofibrils to technical fibers

**DOI:** 10.1617/s11527-022-02097-2

**Published:** 2023-01-12

**Authors:** Markus Königsberger, Markus Lukacevic, Josef Füssl

**Affiliations:** 1 Institute for Mechanics of Materials and Structures, TU Wien, Karlsplatz 13/202, 1040 Vienna, Austria; 2 BATiR Department, Université Libre de Bruxelles, CP194/04, 50 avenue F.D. Roosevelt, 1050 Brussels, Belgium

**Keywords:** Natural fibers, Biocomposite, Strength, Elasticity, Micro-mechanics

## Abstract

The mechanical properties of natural fibers, as used to produce sustainable biocomposites, vary significantly—both among different plant species and also within a single species. All plants, however, share a common microstructural fingerprint. They are built up by only a handful of constituents, most importantly cellulose. Through continuum micromechanics multiscale modeling, the mechanical behavior of cellulose nanofibrils is herein upscaled to the technical fiber level, considering 26 different commonly used plants. Model-predicted stiffness and elastic limit bounds, respectively, frame published experimental ones. This validates the model and corroborates that plant-specific physicochemical properties, such as microfibril angle and cellulose content, govern the mechanical fiber performance.

## Introduction

**Fig. 1:**
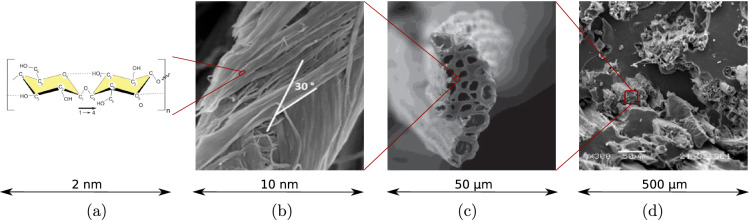
Multiscale structure of biocomposites: **a** physicochemical structure of cellulose chain [14], **b** SEM image of cellulose microfibril in bamboo fiber with microfibril angle of 30$^\circ$ [15], **c** SEM image of jute fiber cross Sect. [10], **d** hemp oil-based biocomposite with jute fiber reinforcements [16]

Increasing environmental concerns have led to a renaissance of natural plant fibers. They are abundantly available in most regions of the world and have proven to be a sustainable and cost-effective alternative to synthetic fibers for the production of high-performance fiber-reinforced biocomposite materials, see Fig. 1d, usable across several engineering fields [1–6], e.g. for lightweight structural elements in the construction sector. Given the sheer amount of possible source materials (including fibers from different plants as well as different binders) and different fiber treatment and composite production technologies, which result in a very specific mechanical composite performance [5, 7–9], micromechanics-based modeling of the three-dimensional mechanical behavior is essential to characterize and optimize existing composites and engineer new ones. As a prerequisite for biocomposites modeling, a micromechanics-based description of the mechanical behavior of plant fibers used for biocomposite production is essential and is dealt with herein.

The mechanical properties of natural fibers vary significantly, ranging from axial moduli of only 10 GPa and axial tensile strengths of less than 100 MPa for coir or oil palm leaf fibers [10, 11] to moduli up to 100 GPa and strengths up to 1800 MPa for bast fibers such as flax or ramie [4, 12, 13], which is comparable to synthetic glass fibers. Even for a given fiber species, the mechanical fiber properties fluctuate, depending on geographical locations, maturity at harvest, location within the plant, growing conditions, processing methods, and potential treatments [2, 5, 6].

Despite the variety, all natural fibers share a common microstructural fingerprint [10], see Fig. 1a–c, which is shortly recalled next. Technical plant fibers typically consist of many fiber cells, formed by the central lumen, surrounded by the cell wall and connected together by the middle lamellae, see Fig. 1c. The cell wall, in turn, consists of a primary and several secondary layers, out of which the S2 layer is by far the thickest [17]. The S2 layer contains amorphous lignin and hemicellulose regions intermixed with cellulose microfibrils disposed in a right-hand spiral, see Fig. 1(b). The angle between the fiber axis and the microfibrils in the S2 layer, denoted as microfibril angle, is a key driver for the mechanical properties of the fiber [1]. The microfibrils themselves contain cellulose either in highly ordered arrangement forming smaller nanofibrils (crystalline cellulose) or disordered arrangement (amorphous cellulose) [14]. The excellent mechanical properties of plant fibers originate from the cellulose nanofibril which, at the molecular scale, is built up by a linear chain of anhydroglucose rings, linked together by covalent oxygen bonds and stabilized by hydrogen bonds [14], see Fig. 1a. The mechanical properties of cellulose nanofibrils themselves have been deciphered recently: the molecular dynamics-derived elastic modulus (in axial chain direction) amounts to roughly 170 GPa [18, 19], the axial tensile strength, in turn, quantified by sonication-induced fragmentation testing [20], amounts to roughly 2300 MPa.

The goal of this paper is to quantitatively link the nanoscale cellulose properties (170 GPa modulus, 2300 MPa strength) to the macroscopic properties of common plant fibers (10–100 GPa modulus, 100–1800 MPa strength). We explore whether both the reduction of the mechanical performance upon transition from the nanoscale to the macroscale as well as the differences in mechanical properties among the fibers from different plants result from plant-specific physicochemical parameters such as microfibril angle, cell wall thickness, and lumen size, as well as from plant-specific amounts and crystallinities of cellulose. As for the required scale transition, we rely on micromechanics-based multiscale modeling. Microstructure-based models for predicting mechanical properties of plant fibers have been developed for several decades. Hearle [21] modeled the plant cell walls as spiral springs. To model the different layers in the cell walls, laminate theory was frequently used [22–24]. More recently, continuum micromechanics multiscale models have been successfully applied to predict the (poro-)elastic stiffness [25–27] and elastic limits [28, 29] of clear-wood. Gangwar and co-workers [30, 31] extended the micromechanics model to predict the axial, shear, as well as bending behavior of whole plant culms, which was in good agreement with experimentally tested behavior of bamboo and oat stems. In this paper, we adopt and extend the continuum micromechanics multiscale representation of wood [25–27] towards the most common natural fibers. Thereby, we consider only the essential microstructural features, i.e. the elongated cylindrical shape of cellulose fibrils and lumen pores, the microfibril angle in the S2 layer, and the hierarchical organization and interaction, as well as the contents of the microstructural constituents.

For model validation, 26 of the most common plant fibers, including fibers obtained from bast, grass, leaves, fruits, seeds, and straws are studied. As for the required model input regarding physicochemical fiber properties, we gather published experimental data, which reveals, much like the mechanical fiber properties, rather significant differences in between different literature sources, resulting in rather large intervals for each property reported in the literature. Notably, measured microscopic physicochemical and macroscopic mechanical fiber properties typically stem from different experimental campaigns. This renders the comparison of model-predicted mechanical properties, which rest on the physicochemical input properties, to experimentally determined counterparts, obtained from single-fiber testing, rather difficult. To overcome this challenge and to avoid any bias, we collect large databases for both the microscopic physicochemical and macroscopic mechanical fiber properties. The micromechanics model is then evaluated for the collected physicochemical interval, whereby we combine (i) unfavorable features such as a small cellulose contents with a large microfibril angles and with a large lumen porosities, to obtain a lower bound for the predicted stiffness and strength; and (ii) favorable features to obtain an upper bound. This results in predicted intervals of macroscopic mechanical fiber properties, which can be justly compared to the corresponding experimentally measured intervals, for all 26 studied fibers. This way, despite all experimental challenges and scattered fiber behavior, the model performance can be assessed and plant-specific differences can be discussed appropriately.

## Multiscale micromechanics modeling

### Micromechanics representation of plant-based biocomposites

The complex microstructure of plant fibers is taken into account by several linked representative volume elements (RVEs), describing the material morphology at different length scales. The material phases defining each RVE are represented by homogeneous sub-domains, whose physical quantities (such as density, stiffness) are intrinsic and known. Notably, RVEs have to satisfy the scale separation criterion [32], reading as

1 $$\begin{aligned} d \ll \ell \ll {\mathcal {L}}\,. \end{aligned}$$

Inequalities (1) imply that the RVE’s characteristic size $\ell$ is considerably larger than the characteristic size *d* of the material phases contained inside the RVE and, at the same time, $\ell$ is considerably smaller than characteristic size ${\mathcal {L}}$ of the structure containing the RVE. In a multiscale setting of RVEs, ${\mathcal {L}}$ takes the role of a phase which is further resolved at a smaller observation scale, i.e. RVE 1 has to be significantly smaller than the phase (inside a larger RVE 2) which is built up by RVE 1.

**Fig. 2:**
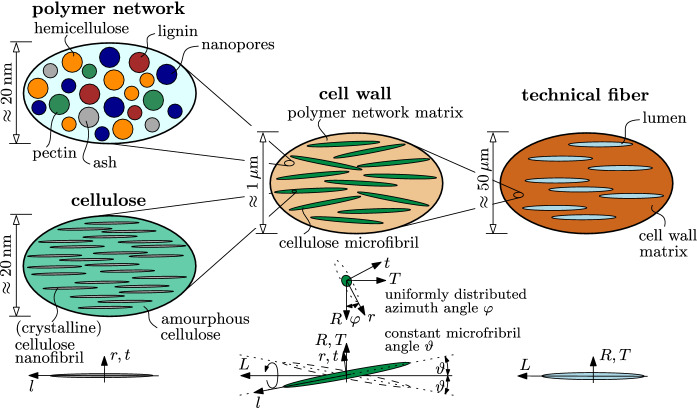
Multiscale micromechanics representation of plant fibers by means of four scale-separated RVEs across three scales of observation, and orientation of phases with respect to local microfibril-related base frame $\underline{e}{}_r,\underline{e}{}_t,\underline{e}{}_l$ or the global base frame $\underline{e}{}_R,\underline{e}{}_T,\underline{e}{}_L$; 2D sketches refer to 3D RVEs

We model plant fibers by means of four RVEs distributed across three scales of observation, as described next. At the scale of several nanometers we adopt the representation of wood, originally developed to predict the elastic properties [25, 26], later extended towards strength predictions [28, 33], poromechanics [27, 29] and towards plant culms [30, 31]. In more detail, an RVE referred to as “polymer network” is considered to consist of five spherical phases: hemicellulose, lignin, pectin, nanopores [initially filled with extractives (including waxes, oils, fats), and potentially emptied during fiber processing], and ashes (inorganic parts), see Fig. 2. At the same length scale, we introduce an that the “cellulose” RVE which is built up by unidirectional crystalline cellulose nanofibril phases, modeled as infinitely long aligned cylinders embedded in a matrix of amorphous cellulose.

At the scale of a single micron, we consider the “cell wall” RVE to consist of infinitely long cylindrical cellulose microfibrils embedded in a matrix of polymer network. The crystalline cellulose nanofibrils are aligned with the cellulose microfibril orientation with coordinate base *r*, *t*, *l*, whereby *l* is the longitudinal direction along the fibril axis. Cellulose microfibrils and plant fibers (with coordinate base *R*, *T*, *L*), however, are typically not aligned. To account for the microfibril orientation, found in the central S2 layer of plant cell walls [34], the microfibrils are considered to be rotated by a constant microfibril angle $\theta$, defined as the angle between the microfibril orientation *l* and the longitudinal plant fiber direction *L*, see the orientations indicated below the RVEs in Fig. 2. The microfibril orientation with respect to the $R-T$-plane is considered to be uniformly distributed. Finally, at the scale of several tens of microns, we consider the RVE of the “technical fiber”, multi-cellular structures with several individual tracheids bound to a fiber bundle [35], which are modeled by a cell wall matrix phase with embedded infinitely long cylindrical lumen porosity aligned with the longitudinal fiber direction *L*.

### Stiffness homogenization

We herein aim at stiffness upscaling, i.e. at homogenizing the stiffness of the micro- or nanoscopic phases to predict, based on the envisioned micromechanics multiscale representation of Fig. 2, the stiffness of the technical fibers from different plants. All phases are considered to be linear elastic and also intrinsic, i.e. they do not vary from one plant to another. Crystalline I$\beta$ cellulose, the dominant polymorph for plants [14], has been studied experimentally by means of X-ray scattering [36–38] and atomic force microscopy [39], as well as numerically by means of molecular simulations [18, 40–42]. The molecular structure of cellulose is characterized by strong covalent bonds in longitudinal *l*-direction (local base) but rather weak bonding by Van der Waals forces in the other directions, resulting in a large longitudinal but rather small radial/tangential stiffness [42]. Reported elastic moduli in longitudinal direction range from 110 GPa [41] to 220 GPa [38], see also the review of Moon et al. [14]. We rely on the well-established simulation results of Tashiro and Kobayashi [18], reporting a modulus of 167.8 GPa, a value close to the center of the reported interval. The corresponding stiffness tensor $\mathbb {C}{}_\text {NF}$ is approximated to be transversally isotropic, with stiffness tensor components, referring to the local microfibril base system $\underline{r}{},\underline{t}{},\underline{l}{}$, reading as [25] [unit: GPa]

2 $$\begin{aligned} \mathbb {C}{}_\text {NF}= \begin{bmatrix} 34.86 &{} 0 &{} 0 &{} 0 &{} 0 &{} 0\\ 0 &{} 34.86 &{} 0 &{} 0 &{} 0 &{} 0\\ 0 &{} 0 &{} 167.8 &{} 0 &{} 0 &{} 0\\ 0 &{} 0 &{} 0 &{} 11.61 &{} 0 &{} 0\\ 0 &{} 0 &{} 0 &{} 0 &{} 11.61 &{} 0\\ 0 &{} 0 &{} 0 &{} 0 &{} 0 &{} 34.86 \end{bmatrix}_{\begin{array}{c} \underline{e}{}_r \\ \underline{e}{}_t \\ \underline{e}{}_l \end{array}}\,, \end{aligned}$$

whereby Kelvin-Mandel tensor notation [43] is used. The remaining phases are considered to be isotropic with phase stiffness tensors $\mathbb {C}{}_i$ reading as

3 $$\begin{aligned} \mathbb {C}{}_i=3\,k_i\mathbb {I}{}_\text {vol}+2\,\mu _i\mathbb {I}{}_\text {dev} \end{aligned}$$

where $k_i$ and $\mu _i$ denote the phase-specific bulk and shear moduli, and are obtained from experiments or molecular models, as summarized in Table 1, and with $\mathbb {I}{}_\text {vol}$ and $\mathbb {I}{}_\text {dev}$ as volumetric and deviatoric parts of the fourth order unity tensor.

**Table 1:** Densities $\rho _i$, as well as bulk moduli $k_i$ and shear moduli $\mu _i$ of phases; properties refer to dry or nearly dry matter

Phase	*i*	$\rho _i$ [g/cm$^3$]	References	$k_i$ [GPa]	$\mu _i$ [GPa]	References
cellulose nanofibril	NF	1.59	[44]	anisotropic, see Eq.(2)		[18]
amorphous cellulose$^\circ$	amcel	1.50	[45]	6.22	2.07	[42]
hemicellulose$^\circ$	hemcel	1.46	[46]	8.08	3.73	[46, 47]
lignin	lig	1.27	[46]	5.00	2.31	[46, 48]
pectin$^*$	pec	1.53	[49]	1	0.4	
nanopores (extractives)	npor	0.9	[25]	0	0	
ash$^\dagger$	ash	2.20	[50]	36.3	30.9	[50]
lumen pores (air-filled)	lum	0		0	0	

$^\circ$Poisson’s ratio assumed to be 0.35 [25]

$^*$elastic properties assumed

$^\dagger$properties of amorphous silica (silica glass) are considered representative

Considering (i) linear constitutive relations ${\varvec{\sigma _i}}=\mathbb {C}{}_i:{\varvec{{\varepsilon }_i}}$ with ${\varvec{{\sigma }_i}}$ and ${\varvec{{\varepsilon }_i}}$ as average phase stresses and strains, (ii) linearized strain-displacement relations, and (iii) equilibrium within an RVE containing *N* perfectly bonded phases, as well as homogeneous boundary conditions, implies a linear strain concentration relation from macrostrains ${\varvec{E}}$ down to microstrains ${\varvec{{\varepsilon }_i}}$, reading as [32, 51]

4 $$\begin{aligned} {\varvec{{\varepsilon }}}_i=\mathbb {A}{}_{i}:{\varvec{E}} \qquad \forall i\in N\,, \end{aligned}$$

and thus a linear stiffness homogenization rule [51]

5 $$\begin{aligned} \mathbb {C}{}_\text {hom}=\sum \limits _{i=1}^N f_i \, \mathbb {C}{}_i : \mathbb {A}{}_i \end{aligned}$$

with $\mathbb {A}{}_i$ as the (fourth-order) phase strain concentration tensor and $f_i$ as the phase volume fraction (satisfying $\sum _{i=1}^{N}f_i=1$). Estimates for $\mathbb {A}{}_i$ in continuum micromechanics, are obtained by introducing *N* Eshelby-type matrix-inclusion problems [52], such that the inclusion in one Eshelby problem represents one spheroidal phase of the RVE. They read as [32]

6 $$\begin{aligned} \mathbb {A}{}_i= \mathbb {A}{}_{i}^0: \left( \sum \limits _{j=1}^N f_j \mathbb {A}{}_{j}^0\right) ^{-1}\,, \end{aligned}$$

with auxiliary concentration tensors $\mathbb {A}{}_{i}^0$ reading as

7 $$\begin{aligned} \mathbb {A}{}_{i}^0=\left[ \mathbb {I}{}+\mathbb {P}{}_i:\left( \mathbb {C}{}_i-\mathbb {C}{}_0\right) \right] ^{-1}\,, \end{aligned}$$

whereby $\mathbb {I}{}$ is the (fourth-order) identity, $\mathbb {P}{}_i$ is the (fourth-order) Hill tensor accounting for the inclusion shape (see A), and $\mathbb {C}{}_0$ is the stiffness of the infinite matrix in the Eshelby problem and is chosen based on the mode of interaction between the phases inside the RVE.

Stiffness homogenization according to Eqs. (5–7) is applied to the RVEs depicted in Fig. 2, starting at the smallest observation scale. The homogenized stiffness of the polymer network $\mathbb {C}{}_\text {pn}$ follows from specialization of homogenization Eqs. (5–7) for $N=5$ spherical and isotropic phases for mutual interactions between all phases considered by the self-consistent scheme [53, 54] as

8 $$\begin{aligned} \begin{aligned} \mathbb {C}{}_\text {pn}=&\left[ \sum _{i}f_i^\text {pn}\,\mathbb {C}{}_i: \left[ \mathbb {I}{}+\mathbb {P}{}_\text {sph}^\text {pn}:\left( \mathbb {C}{}_i-\mathbb {C}{}_\text {pn}\right) \right] ^{-1} \right] : \\&\left[ \sum _{i}f_i^\text {pn} \left[ \mathbb {I}{}+\mathbb {P}{}_\text {sph}^\text {pn}:\left( \mathbb {C}{}_i-\mathbb {C}{}_\text {pn}\right) \right] ^{-1} \right] ^{-1} \end{aligned} \end{aligned}$$

with $i\in {\{\text {hemcel,lig,pec,wax,ash}\}}$ and $\mathbb {P}{}_\text {sph}^\text {pn}$ as the Hill tensor of spherical phases in an infinite matrix of polymer network, see A. The homogenized stiffness of the cellulose microfibril $\mathbb {C}{}_\text {MF}$ follows from specialization of homogenization Eqs. (5–7) for $N=2$ phases and for the envisioned matrix-inclusion morphology modeled by the Mori-Tanaka homogenization scheme [55, 56] as

9 $$\begin{aligned} \begin{aligned} \mathbb {C}{}_\text {MF}=&\left( f_\text {amcel}^\text {cel}\,\mathbb {C}{}_\text {amcel}+ f_\text {NF}^\text {cel}\,\mathbb {C}{}_\text {NF}:\mathbb {A}{}_\text {NF}^0 \right) :\\&\left( f_\text {amcel}^\text {cel}\mathbb {I}{}+f_\text {NF}^\text {cel} \mathbb {A}{}_\text {NF}^0 \right) ^{-1} \end{aligned} \end{aligned}$$

with

10 $$\begin{aligned} \mathbb {A}{}_\text {NF}^0=\left[ \mathbb {I}{}+\mathbb {P}{}_\text {cyl}^\text {amcel} :\left( \mathbb {C}{}_\text {NF}-\mathbb {C}{}_\text {amcel}\right) \right] ^{-1}\,, \end{aligned}$$

and with Hill tensor components of $\mathbb {P}{}_\text {cyl}^\text {pn}$ given in A. The homogenized stiffness of the cell wall $\mathbb {C}{}_\text {cw}$ follows from specialization of homogenization Eqs. (5–7) for $N=2$ phases, and again for the envisioned matrix-inclusion morphology modeled by the Mori-Tanaka homogenization scheme [55, 56]. Given the microfibril orientation with constant microfibril angle $\vartheta \ge 0$ but uniform orientation along the azimuth $\varphi$, integration along the circumference is required, yielding

11 $$\begin{aligned} \begin{aligned} \mathbb {C}{}_\text {cw}=&\left[ f_\text {pn}^\text {cw}\,\mathbb {C}{}_\text {pn}+ \frac{f_\text {MF}^\text {cw}}{2\pi } \int \limits _0^{2\pi }\mathbb {C}{}_\text {MF}:\mathbb {A}{}_\text {MF}^0(\varphi )\, \textrm{d}\varphi \right] : \\&\left[ f_\text {pn}^\text {cw}\mathbb {I}{}+ \frac{f_\text {MF}^\text {cw}}{2\pi } \int \limits _0^{2\pi }\mathbb {C}{}_\text {MF}:\mathbb {A}{}_\text {MF}^0(\varphi )\, \textrm{d}\varphi \right] ^{-1} \end{aligned} \end{aligned}$$

with

12 $$\begin{aligned} \mathbb {A}{}_\text {MF}^0(\varphi )=\left[ \mathbb {I}{}+\mathbb {P}{}_\text {cyl}^\text {pn}(\varphi ) :\left( \mathbb {C}{}_\text {MF}-\mathbb {C}{}_\text {pn}\right) \right] ^{-1}\,, \end{aligned}$$

with Hill tensor components of $\mathbb {P}{}_\text {cyl}^\text {pn}$ given in A. Notably, any asymmetries related to Mori-Tanaka homogenization with anisotropic phases [57] are symmetrized [58]. Finally, the homogenized stiffness of the technical fiber $\mathbb {C}{}_\text {fib}$ follows from specialization of homogenization Eqs. (5–7) for $N=2$ phases and again for the envisioned matrix-inclusion morphology modeled by the Mori-Tanaka homogenization scheme [55, 56] as

13 $$\begin{aligned} \mathbb {C}{}_\text {fib}= f_\text {cw}^\text {fib}\,\mathbb {C}{}_\text {cw}: \left[ f_\text {cw}^\text {cel}\mathbb {I}{}+f_\text {lum}^\text {cel} \left( \mathbb {I}{}-\mathbb {P}{}_\text {cyl}^\text {cw}:\mathbb {C}{}_\text {cw}\right) ^{-1} \right] ^{-1} \end{aligned}$$

with Hill tensor components of $\mathbb {P}{}_\text {cyl}^\text {cw}$ given in A. The sought axial fiber modulus $E_\text {fib}=1/D_{\text {fib},LLLL}$, with $\mathbb {D}{}_\text {fib}=\mathbb {C}{}_\text {fib}^{-1}$ as the fiber compliance tensor.

### Elastic limit homogenization

Cellulose failure is considered to be responsible for failure of natural fibers. Experimental insights into cellulose failure is therefore discussed first. Access to the tensile failure properties of cellulose fibrils is currently limited to sonication-induced fragmentation testing from Saito et al. [20] and Lee et al. [59]. Native cellose nanofibrils were isolated by means of TEMPO-mediated oxidation, and the suspensions were subsequently subjected to hydrodynamic stresses through sonication-induced cavitation, yielding fibril fragmentation. After prolonged sonication treatment, remaining fibrils exhibit lengths smaller than a threshold length, from which a tensile strength estimate can be deduced. The arithmetic mean strength of wood cellulose nanofibrils, which are herein considered representative for all plant fibers, and which exhibit diameters of 3 nm (as measured from X-ray diffraction), amount to 2.3 GPa [20]. Notably, the tensile strength of crystalline 1$\beta$ cellulose, the dominant polymorph for higher-plant cell wall cellulose [14], is even two to three times higher than the reported nanofibril strength, as revealed by means of molecular dynamics [60] of defect-free cellulose. Cellulose nanofibrils with lengths of several hundred nanometers, as the ones tested by Saito et al. [20], however, exhibit defects and/or may contain thin amorphous regions [14], such that the intrinsic strength of crystalline 1$\beta$ cellulose is not reached. In our model, we assume that the nanofibril strength amounts to ${\sigma }_{\text {NF},ll}^\text {ult}=2.3$ GPa, as this value already accounts for interfaces/defects present in nanofibrils. The defects, which represent localized weaknesses/breaking points, are not expected to alter density or stiffness of the nanofibrils significantly. Therefore, the properties of crystalline cellulose, given in Table 1 and Eq. (2), are still valid for the nanofibrils.

**Fig. 3:**
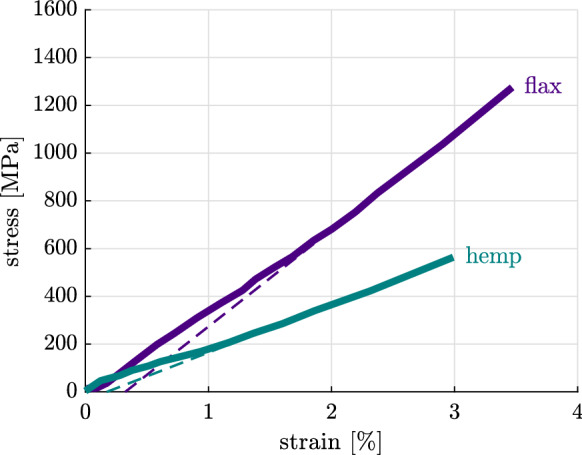
Experimental stress-strain relation (continuous lines) obtained from single fiber testing [62]; the dashed lines are a linear extrapolation of the final slope revealing nonlinearities only at small stress levels

In this paper we test whether the experimentally determined cellulose nanofibril strength can be upscaled to elastic limits of technical fibers from several different plants. Single fiber tensile tests [13, 61] indicate that stress-strain relations are virtually linear followed by brittle rupture. Marrot et al. [62] observed some minor pre-peak nonlinearities at low stress levels, see Fig. 3, which can be explained by the progressive alignment of the microfibrils with the fiber axis [63].

Moreover, elasto-brittle failure is also observed in molecular simulations of crystalline cellulose [60].

Given the quasi-brittle failure of cellulose-based fibers and the limited experimental insight, we aim at an engineering approach and consider that the fiber’s elastic limit is equal to the fiber strength and that it follows from brittle failure of cellulose nanofibrils. We note that similar elasto-brittle approaches have been very successfully applied to predict the elastic limits and strengths of various composite materials, including cement paste [64], concrete [65], wood [28], plant culms [31] bone [66], or shale [67]. In more detail, we consider that the technical plant fiber remains intact as long as the longitudinal stress of the crystalline cellulose nanofibrils ${\sigma }_{\text {NF},ll}$, obtained from elastic stress concentrations, is smaller than the experimentally determined tensile strength ${\sigma }_{\text {NF},ll}^\text {ult}$, which can be mathematically expressed by the failure function ${\mathcal {F}}_\sigma$ as

14 $$\begin{aligned} {\mathcal {F}}_\sigma ={{\sigma }}_{\text {NF},ll}-{\sigma }_{\text {NF},ll}^\text {ult}\le 0\,, \end{aligned}$$

Cellulose nanofibril stresses ${\varvec{{\sigma }}}_\text {NF}$ are obtained from downscaling the macroscopic uniaxial tensile loading ${\varvec{{\Sigma }}}={\Sigma }\,\underline{e}{}_L \otimes \underline{e}{}_L$, with ${\Sigma }>0$ as macroscopic tensile stress. Therefore, the macrostresses are first translated to macrostrains by applying the inverse form of the generalized Hooke’s law ${\varvec{E}}=\mathbb {C}{}_\text {fib}^{-1}:{\varvec{{\Sigma }}}$, then average phase strains are obtained from step-wise strain downscaling according to the strain concentration relations (4), (6), and (7), and finally cellulose nanofibril phase stresses are obtained by Hooke’s law ${\varvec{{\sigma }}}_\text {NF}=\mathbb {C}{}_\text {NF}:{\varvec{{\varepsilon }}}_\text {NF}$, yielding

15 $$\begin{aligned} \begin{aligned}&{\varvec{{\sigma }}}_{\text {NF}}(\varphi ) \\&\quad =\mathbb {C}{}_\text {NF}: \mathbb {A}{}_\text {NF}^0:\left( f_\text {amcel}^\text {cel}\mathbb {I}{}+f_\text {NF}^\text {cel} \mathbb {A}{}_\text {NF}^0\right) ^{-1}:\mathbb {A}{}_\text {MF}^0(\varphi ) \\&\quad : \left[ f_\text {pn}^\text {cw}\mathbb {I}{}+ \frac{f_\text {MF}^\text {cw}}{2\pi } \int \limits _0^{2\pi }\mathbb {C}{}_\text {MF}:\mathbb {A}{}_\text {MF}^0(\varphi )\, \textrm{d}\varphi \right] ^{-1} \\&\quad : \left[ f_\text {cw}^\text {cel}\mathbb {I}{}+f_\text {lum}^\text {cel} \left( \mathbb {I}{}-\mathbb {P}{}_\text {cyl}^\text {cw}:\mathbb {C}{}_\text {cw}\right) ^{-1} \right] ^{-1}:\mathbb {C}{}_\text {fib}^{-1} \\&\quad : {\Sigma }\,\underline{e}{}_L \otimes \underline{e}{}_L\,. \end{aligned} \end{aligned}$$

Notably, the longitudinal nanofibril stress component ${\varvec{{\sigma }}}_{\text {NF},ll}$, which governs tensile failure according to failure function (14), is constant with respect to the nanofibril’s azimuth orientation $\varphi$. The sought axial fiber tensile elastic limit ${\Sigma }_\text {fib}^\text {ult}$ corresponds to the macrostress magnitude ${\Sigma }$ for which failure criterion (14), evaluated for nanofibril stresses ${\varvec{{\sigma }}}_\text {NF}$ according to Eq. (15), becomes zero.

### Plant-specific standard fiber properties and phase volume fractions

Plant-specific physicochemical fiber properties are reported herein, and corresponding phase volume fractions are derived. We focus on 26 of the most common plant fibers reported in the literature, grouped into five fiber types: bast (banana, fiber flax, hemp, isora, jute, kenaf, ramie, sorghum), grass (alfa, bagasse, bamboo), leaf (abaca, curaua, henequen, phormium/harekeke, pineapple, sisal), fruits or seeds (coir, kapok, oil palm), and straw (barley, cornhusk, cornstalk, rice, soybean, wheat). Physicochemical fiber properties depend on the plant species, geographical location, growing conditions, the maturity at harvesting, the exact fiber location within the plant, the fiber extraction process, potential alkali treatment, storage conditions, and several factors related to the testing procedure [5, 9, 68]. To capture this variety, we collect physicochemical properties from several different sources, and report on intervals. In more detail, minima, representative averages, and maxima for all properties are reported, see Tables 2 and 3, as discussed next.

**Table 2:** Microscopic physicochemical composition in terms of cell wall-related mass fractions $\tilde{m}_i^\text {cw}$ in percent (fractions are not normalized): minimum/**representative average**/maximum values are reported as collected from published experimental data

Name	Type	Total cell.	Hemicell.	Lignin	Pectin	Ash	Nanopore	References
Banana	B	60/**71**/82	6/**10**/14	5/**8**/10	0/**4**/4	0/**5**/5	0/**0**/0	[9, 69, 70]
Flax	B	71/**73**/81	15/**18**/21	2/**3**/3	–/**1**/–	–/**0**/–	–/**2**/–	[9, 68, 71]
Hemp	B	57/**70**/81	18/**20**/22	4/**5**/6	–/**1**/–	–/**0**/–	–/**1**/–	[68, 72, 73]
Isora	B	71/**71**/75	0/**3**/3	14/**21**/23	–/**0**/–	0/**0**/1	–/**0**/–	[9, 74, 75]
Jute	B	45/**58**/72	12/**18**/23	9/**18**/26	0/**0**/0	–/**0**/–	–/**0**/–	[9, 68, 72]
Kenaf	B	31/**56**/81	–/**22**/–	15/**17**/19	–/**2**/–	–/**0**/–	–/**0**/–	[9, 68, 72]
Ramie	B	69/**80**/91	5/**14**/17	1/**1**/1	–/**2**/–	–/**0**/–	–/**0**/–	[9, 68, 72]
Sorghum	B	–/**65**/–	–/**19**/–	–/**10**/–	–/**0**/–	–/**5**/–	–/**0**/–	[76]
Alfa	G	–/**45**/–	–/**39**/–	–/**39**/–	–/**0**/–	–/**0**/–	–/**2**/–	[9]
Bagasse	G	32/**37**/48	19/**23**/28	23/**7**/32	0/**10**/10	2/**4**/5	0/**2**/4	[9, 69]
Bamboo	G	35/**35**/47	15/**21**/23	21/**26**/31	0/**0**/0	–/**0**/–	–/**0**/–	[9, 77]
Abaca	L	56/**63**/70	14/**20**/25	7/**9**/12	0/**1**/1	0/**2**/2	3/**6**/6	[9, 68, 78]
Curaua	L	–/**74**/–	–/**10**/–	–/**8**/–	–/**0**/–	–/**0**/–	–/**1**/–	[79]
Henequen	L	60/**64**/68	18/**23**/28	8/**8**/9	–/**0**/–	0/**1**/1	–/**5**/–	[9, 78]
Phormium	L	–/**67**/–	–/**30**/–	–/**11**/–	–/**0**/–	–/**0**/–	–/**0**/–	[9]
Pineapple	L	70/**75**/85	–/**18**/–	5/**8**/12	0/**4**/4	1/**1**/1	–/**0**/–	[9, 69, 80]
Sisal	L	38/**63**/88	10/**14**/26	8/**12**/25	0/**10**/10	–/**0**/–	–/**3**/–	[69, 72, 80, 81]
Coir	F	21/**40**/46	12/**20**/31	20/**35**/47	0/**4**/4	0/**10**/10	0/**0**/9	[9, 69]
Kapok	F	53/**59**/64	29/**30**/30	13/**17**/22	–/**0**/–	1/**1**/1	–/**4**/–	[82]
Oil Palm	F	43/**54**/65	17/**25**/34	13/**19**/25	0/**0**/0	1/**4**/6	–/**7**/–	[11]
Barley	S	31/**38**/45	27/**33**/38	14/**17**/19	–/**0**/–	–/**0**/–	–/**0**/–	[69]
Cornhusk	S	47/**54**/61	20/**32**/44	2/**3**/4	–/**0**/–	3/**8**/13	–/**0**/–	[83]
Cornstalk	S	38/**39**/40	–/**28**/–	7/**14**/21	–/**0**/–	4/**0**/7	–/**0**/–	[9, 69]
Rice	S	28/**32**/36	23/**26**/28	12/**13**/14	–/**0**/–	14/**17**/20	–/**0**/–	[69]
Soybean	S	35/**85**/88	0/**5**/17	5/**11**/22	–/**0**/–	1/**2**/11	–/**0**/–	[84, 85]
Wheat	S	33/**36**/38	26/**29**/32	17/**18**/19	–/**0**/–	2/**0**/7	–/**0**/–	[69]

**Table 3:** Microscopic physicochemical fiber properties related to the minimum/**representative average**/maximum homogenized fiber stiffness/strength: reported microfibril angle, reported volumetric crystallinity, reported fiber densities, calculated cell wall density according to Eq. (26), and calculated lumen porosity according to Eq. (25)

Name	Type	Microfibril angle		Crystallinity		Density [g/cm$^3$]			lumen
		$\vartheta$ [$^\circ$]	References	$\xi _V$ [%]	References	$\rho _{fib}$	References	$\rho _{cw}$	$f_{lpor}^{fib}$ [$\%$]
Banana	B	11/**11**/12	[86, 87]	30/**45**/49	[88, 89]	1.3/**1.4**/1.5	[86]	**1.51**	14/**7**/0
Flax	B	5/**6**/10	[1, 86]	50/**78**/90	[71, 87, 90, 91]	1.38/**1.38**/1.5	[86, 92]	**1.48**	7/**7**/0
Hemp	B	6/**7**/10	[1, 86, 93]	50/**75**/98	[73, 87, 91, 94]	1.35/**1.4**/1.5	[86, 92, 93]	**1.48**	9/**6**/0
Isora	B	20/**23**/26	[74]	34/**71**/71	[74, 95]	1.2/**1.3**/1.3	[12, 96]	**1.44**	16/**9**/9
Jute	B	7/**8**/9	[1, 86, 93]	68/**71**/73	[89, 97, 98]	1.23/**1.35**/1.5	[86, 89, 92, 93]	**1.42**	13/**5**/0
Kenaf	B	9/**12**/15	[71, 93]	61/**65**/69	[71]	1.2/**1.22**/1.4	[92, 93]	**1.45**	17/**16**/3
Ramie	B	6/**8**/10	[93]	–/**64**/-	[87]	1.44/**1.44**/1.55	[86, 92, 93]	**1.51**	4/**4**/0
Sorghum	B	16/**16**/17	[99]	32/**43**/53	[98–100]	–/**0.89**/-	[101]	**1.5**	47/**41**/35
Alfa	G	–/**10**/–	$^*$	–/**64**/–	[102]	–/**0.89**/–	[12]	**1.41**	43/**37**/30
Bagasse	G	14/**15**/15	[87]	–/**48**/–	[35]	0.55/**1.2**/1.25	[86, 92]	**1.34**	59/**10**/7
Bamboo	G	2/**6**/10	[93]	40/**56**/60	[87, 90]	1.2/**1.3**/1.5	[103]	**1.29**	7/**0**/0
Abaca	L	–/**23**/–	[87]	–/**52**/–	[87]	–/**1.5**/–	[86, 93]	**1.45**	7/**0**/0
Curaua	L	15/**17**/19	[79]	–/**66**/–	[79]	–/**1.4**/–	[12]	**1.44**	12/**3**/0
Henequen	L	18/**20**/22	[93]	44/**47**/50	[104]	1.33/**1.4**/1.4	[92, 93]	**1.45**	8/**3**/3
Phormium	L	–/**10**/–	$^*$	–/**69**/–	[105]	–/**1.27**/–	[106]	**1.5**	24/**15**/7
Pineapple	L	6/**12**/14	[1, 86, 87]	44/**52**/60	[88]	1.32/**1.5**/1.56	[12, 86, 92]	**1.51**	13/**1**/0
Sisal	L	10/**20**/25	[1, 86, 87]	68/**73**/77	[107]	1.2/**1.35**/1.5	[86, 89, 92, 93]	**1.47**	19/**8**/0
Coir	F	30/**45**/49	[1, 86, 93]	27/**30**/33	[87]	1.2/**1.2**/1.25	[2, 86, 89]	**1.46**	18/**18**/14
Kapok	F	–/**10**/–	$^*$	–/**46**/–	[107]	–/**0.38**/–	[86]	**1.44**	76/**73**/71
Oil Palm	F	–/**46**/–	[87]	20/**25**/30	[108]	0.7/**1.35**/1.55	[87]	**1.41**	50/**4**/0
Barley	S	–/**10**/–	$^*$	–/**50**/–	$^*$	–/**0.52**/–	$^*$	**1.35**	65/**61**/58
Cornhusk	S	–/**10**/–	$^*$	48/**74**/100	[88, 94]	0.43/**0.52**/0.61	[109]	**1.52**	72/**66**/60
Cornstalk	S	–/**11**/–	[110]	52/**76**/100	[87, 94]	–/**0.52**/–	$^*$	**1.31**	64/**60**/56
Rice	S	–/**10**/–	$^*$	40/**60**/63	[88, 98]	–/**1.65**/–	[87]	**1.43**	0/**0**/0
Soybean	S	–/**12**/–	[87]	43/**47**/51	[84, 87]	–/**0.52**/–	$^*$	**1.51**	69/**66**/62
Wheat	S	–/**0**/–	[111]	48/**51**/51	[71, 90]	1.45/**1.53**/1.6	[87]	**1.31**	0/**0**/0

$$^*$$Assumed

Average cell wall-related phase volume fractions are derived first, for which physicochemical composition and cellulose crystallinity are discussed next. The cell wall composition of plant fibers has been studied extensively by means of several physicochemical analysis methods, such as acid hydrolysis, chromatography, Klason lignin analysis, and thermogravimetric analysis [112]. This way, (minimum, representative average, and maximum) cell wall-related mass fractions $\tilde{m}_i^\text {cw}$ of (total = crystalline + amorphous) cellulose, hemicellulose, lignin, pectin, ash, and nanoporosity (wax/fat) were measured and typical results are collected in Table 2. Notably, the reported differences between the representative average and the minimum and maximum values are typically smaller than a few percent. If the sum of average mass fractions exceeds 100 %, phase mass fractions are reduced proportionally through

16 $$\begin{aligned} \begin{aligned} m_i^\text {cw}=&\frac{\tilde{m}_i^\text {cw}}{\sum _j \tilde{m}_i^\text {cw}} \\&i\in \{\text {totcel,hemcel,lig,pec,ash,npor}\}\,. \end{aligned} \end{aligned}$$

If the sum of the average mass fractions is below 100 %, we consider unassigned matter, together with the measured wax/fat mass, as extractive and thus part of the nanoporosity:

17 $$\begin{aligned} m_\text {npor}^\text {cw}=1-m_\text {totcel}^\text {cw}-m_\text {hemcel}^\text {cw}-m_\text {lig}^\text {cw} -m_\text {pec}^\text {cw}-m_\text {ash}^\text {cw}\,. \end{aligned}$$

The cellulose crystallinity is typically given in terms of a volumetric crystallinity index $\xi _V$ derived from XRD spectra [94]. While accurate values depend on the evaluation method [113], we again report minimum, average, and maximum crystallinity indexes (corresponding to the targeted minimum, average, and maximum prediction) found in the literature, see Table 3. The volumetric crystallinity is related to a crystallinity index by mass, $\xi _M$, through

18 $$\begin{aligned} \xi _M=\frac{1}{1+\left( \frac{1}{\xi _V}-1\right) \frac{\rho _\text {amcel}}{\rho _\text {NF}}}\,, \end{aligned}$$

with phase densities $\rho _\text {amcel}$ and $\rho _\text {NF}$ from Table 1. Cell wall-related mass fraction of crystalline ($m_\text {NF}^\text {cw}$) and amorphous ($m_\text {amcel}^\text {cw}$) cellulose, respectively, then follow from the total cellulose mass fraction $m_\text {totcel}^\text {cw}$ given in Table 2 and from crystallinity indices $\xi _M$ according to Eq. (18) as

19 $$\begin{aligned} m_\text {NF}^\text {cw}=\xi _M\,m_\text {totcel}^\text {cw}\,, \qquad m_\text {amcel}^\text {cw}=\left( 1-\xi _M\right) m_\text {totcel}^\text {cw}\,. \end{aligned}$$

Corresponding average phase volume fractions for all constituents of the cell walls then read as

20 $$\begin{aligned} \begin{aligned} f_{i}^\text {cw}=&\frac{m_{i}^\text {cw}/\rho _i}{\sum _j m_{j}^\text {cw}/\rho _j} \\&i,j\in \{\text {NF,amcel,hemcel,lig,pec,ash,npor}\}\,, \end{aligned} \end{aligned}$$

with phase densities $\rho _i$ given in Table 1. Average phase volume fractions for the 26 plants are given as bold values in Table 4.

**Table 4:** Calculated cell wall-related phase volume fractions $f_{i}^\text {cw}$ in percent according to Eq. (20) related to the minimum/**representative average**/maximum homogenized fiber stiffness/strength

Name	Type	Cry. cell.	Am. cell.	Hemicell.	Lignin	Pectin	Ash	Nanopore
Banana	B	18/**31**/40	41/**38**/40	14/**10**/6	13/**10**/6	5/**4**/2	5/**3**/2	5/**3**/2
Flax	B	32/**54**/70	32/**15**/8	21/**18**/13	4/**4**/3	1/**1**/1	0/**0**/0	10/**8**/6
Hemp	B	27/**50**/76	27/**17**/2	28/**20**/13	8/**6**/4	1/**1**/1	0/**0**/0	9/**7**/4
Isora	B	21/**46**/54	40/**19**/17	3/**3**/2	27/**24**/20	0/**0**/0	0/**0**/0	9/**8**/7
Jute	B	26/**37**/51	14/**16**/16	22/**18**/12	26/**20**/14	0/**0**/0	0/**0**/0	12/**9**/7
Kenaf	B	17/**34**/55	11/**18**/23	33/**22**/10	29/**19**/9	3/**2**/1	0/**0**/0	7/**5**/2
Ramie	B	39/**49**/62	27/**28**/28	22/**14**/7	2/**1**/1	3/**2**/1	0/**0**/0	8/**5**/2
Sorghum	B	19/**27**/36	40/**36**/32	22/**20**/17	13/**12**/10	0/**0**/0	4/**3**/3	2/**2**/1
Alfa	G	17/**21**/26	11/**12**/11	32/**30**/28	37/**35**/32	0/**0**/0	0/**0**/0	3/**3**/2
Bagasse	G	12/**15**/23	16/**17**/20	23/**21**/18	8/**7**/6	9/**9**/7	3/**2**/2	30/**28**/24
Bamboo	G	10/**16**/24	15/**13**/16	20/**19**/16	28/**26**/22	0/**0**/0	0/**0**/0	27/**26**/22
Abaca	L	24/**30**/38	27/**28**/28	23/**20**/16	12/**10**/8	1/**1**/1	2/**1**/1	11/**10**/8
Curaua	L	38/**45**/53	24/**23**/21	12/**10**/8	11/**9**/7	0/**0**/0	0/**0**/0	15/**13**/11
Henequen	L	23/**28**/34	32/**32**/31	26/**23**/20	10/**9**/8	0/**0**/0	1/**1**/1	9/**8**/7
Phormium	L	34/**41**/47	20/**19**/17	32/**29**/25	14/**12**/11	0/**0**/0	0/**0**/0	0/**0**/0
Pineapple	L	28/**36**/50	36/**33**/34	20/**18**/9	10/**9**/5	4/**4**/2	1/**1**/0	0/**0**/0
Sisal	L	23/**42**/67	11/**16**/19	22/**14**/5	21/**14**/4	15/**9**/3	0/**0**/0	8/**5**/2
Coir	F	5/**11**/15	15/**25**/29	23/**18**/16	46/**37**/32	4/**3**/3	7/**6**/5	0/**0**/0
Kapok	F	18/**23**/31	26/**27**/30	29/**27**/21	19/**17**/14	0/**0**/0	1/**1**/0	6/**6**/4
Oil Palm	F	8/**11**/18	32/**34**/43	25/**22**/16	22/**19**/14	0/**0**/0	3/**2**/2	11/**10**/7
Barley	S	12/**17**/22	15/**17**/18	34/**31**/28	20/**18**/16	0/**0**/0	0/**0**/0	20/**18**/16
Cornhusk	S	22/**39**/59	24/**14**/0	38/**33**/29	4/**4**/3	0/**0**/0	6/**6**/5	6/**5**/4
Cornstalk	S	15/**25**/37	14/**8**/0	27/**25**/24	15/**15**/14	0/**0**/0	0/**0**/0	29/**28**/26
Rice	S	10/**18**/22	15/**12**/12	27/**26**/24	16/**15**/14	0/**0**/0	12/**11**/10	20/**19**/18
Soybean	S	14/**38**/45	19/**43**/42	18/**5**/3	45/**13**/9	0/**0**/0	5/**1**/1	0/**0**/0
Wheat	S	12/**16**/20	14/**15**/15	28/**26**/24	20/**19**/17	0/**0**/0	0/**0**/0	26/**25**/23

Next, cell wall-related volume fractions associated to both minimum and maximum model predictions, are derived. The maximum fiber stiffness and the maximum fiber strength are obtained for a maximum crystalline cellulose content. This way, we consider that the (normalized) total cellulose mass fraction $m_\text {totcel}^\text {cw}$ is equal to the maximum reported mass fractions $\tilde{m}_\text {totcel}^\text {cw}$ from Table 2, but at least five percentage points larger than the average cellulose mass fractions. The corresponding mass fractions of all other phases (hemicellulose, lignin, pectin, ash, nanopores) related to the maximum case are obtained by proportionally decreasing the average values. The cellulose crystallinities $\xi _V$ for the maximum stiffness/strength case are considered to be equal to the maximum reported cellulose crystallinities from Table 3, but at least five percentage points larger than the average crystallinities. The corresponding phase volume fractions are then calculated through re-evaluation of (18)-(20), see Table 4 for numeric values for all 26 plants. By analogy, the minimum stiffness case relates to minimum reported cellulose mass fractions (but at least five percentage points smaller than their averages) and to minimum reported crystallinities (but at least five percentage points smaller than their averages), see again Table 4 for numeric values.

Volume fractions related to minimum/average/maximum mechanical fiber properties are next assigned to the specific RVEs depicted in Fig. 2. Cell wall-related volume fractions of polymer network ($f_\text {pn}^\text {cw}$) and of the cellulose microfibrils ($f_\text {MF}^\text {cw}$) read as

21 $$\begin{aligned} f_\text {pn}^\text {cw}&=f_\text {hemcel}^\text {cw}+f_\text {lig}^\text {cw}+f_\text {pec}^\text {cw} +f_\text {ash}^\text {cw}+f_\text {npor}^\text {cw}\,, \end{aligned}$$

22 $$\begin{aligned} f_\text {MF}^\text {cw}&=f_\text {NF}^\text {cw}+f_\text {amcel}^\text {cw}\,. \end{aligned}$$

Polymer network-related volume fractions of hemicellulose ($f_\text {hemcel}^\text {pn}$), lignin ($f_\text {lig}^\text {pn}$), pectin ($f_\text {pec}^\text {pn}$), ash ($f_\text {ash}^\text {pn}$), and nanoporosity ($f_\text {npor}^\text {pn}$) read as

23 $$\begin{aligned} f_{i}^\text {pn}=\frac{f_{i}^\text {cw}}{f_\text {pn}^\text {cw}}\,, \quad i\in \{\text {hemcel,lig,pec,ash,npor}\}\,, \end{aligned}$$

and cellulose-related volume fractions of crystalline ($f_\text {NF}^\text {cel}$) and amorphous ($f_\text {amcel}^\text {cel}$) cellulose read as

24 $$\begin{aligned} f_{i}^\text {cel}=\frac{f_{i}^\text {cw}}{f_\text {MF}^\text {cw}}\,, \quad i\in \{\text {NF,amcel}\}\,. \end{aligned}$$

Next, lumen volume fractions at the fiber scale, $f_\text {lum}^\text {cw}$, are derived. Experimentally determined lumen porosities are reported only for a few plant fibers, see e.g. SEM image-based results [114] or density-based results [107]. As a remedy, we back-calculate the lumen porosities from the fiber densities $\rho _\text {fib}$, which are widely reported in the literature, see Table 3 for corresponding minima, averages, and maxima. As for plants, for which only one single density value is found, we consider intervals of ±10 % around the reported value, to quantify the maximum and minimum, respectively. Considering that the fiber density is the product of cell wall density $\rho _\text {cw}$ and fiber-related cell wall volume fraction $f_\text {cw}^\text {fib}$, $\rho _\text {fib}=\rho _\text {cw}\,f_\text {cw}^\text {fib}$ and that $f_\text {lum}^\text {fib}+f_\text {cw}^\text {fib}=1$ allows for deriving the fiber-related volume fractions $f_\text {lum}^\text {fib}$ and $f_\text {cw}^\text {fib}$ as

25 $$\begin{aligned} f_\text {cw}^\text {fib}=\frac{\rho _\text {fib}}{\rho _\text {cw}} \le 1\,, \quad f_\text {lum}^\text {fib}=1-\frac{\rho _\text {fib}}{\rho _\text {cw}}\ge 0\,, \end{aligned}$$

with composition-dependent cell wall density reading as

26 $$\begin{aligned} \begin{aligned} \rho _\text {cw} =&\sum _i \rho _{i}\,f_{i}^\text {cw}\,, \\&i\in \{\text {NF,amcel,hemcel,lig,pec,ash,npor}\}\,. \end{aligned} \end{aligned}$$

Eq. (26) is specialized for average phase volume fractions only, such that the resulting cell wall density is an average quantity, see Table 3. Minimum, average, and maximum fiber-related volume fractions, respectively, then follow from evaluating Eq. (25) with average cell wall densities $\rho _\text {cw}$, but with reported minimum, average, and maximum densities, respectively, see Table 3 for corresponding lumen porosities for all 26 plants. Note that the smallest lumen porosity is related to the maximum stiffness/strength, and vice versa.

Finally, we report on microfibril angles of the 26 plants introduced in the RVEs of Fig. 2. They range from zero to $49^\circ$, see Table 3. If minimum and maximum values are not reported in the database, we assume a range of $\pm 3 ^\circ$ from the reported value. Notably, maximum (or minimum) microfibril angles, yield minimum (or maximum) macroscopic fiber moduli $E_\text {fib}$ as well as minimum (or maximum) macroscopic fiber strength ${\Sigma }_\text {fib}^\text {ult}$.

## Comparison of model-predicted and experimentally measured fiber stiffness/strength intervals

**Fig. 4:**
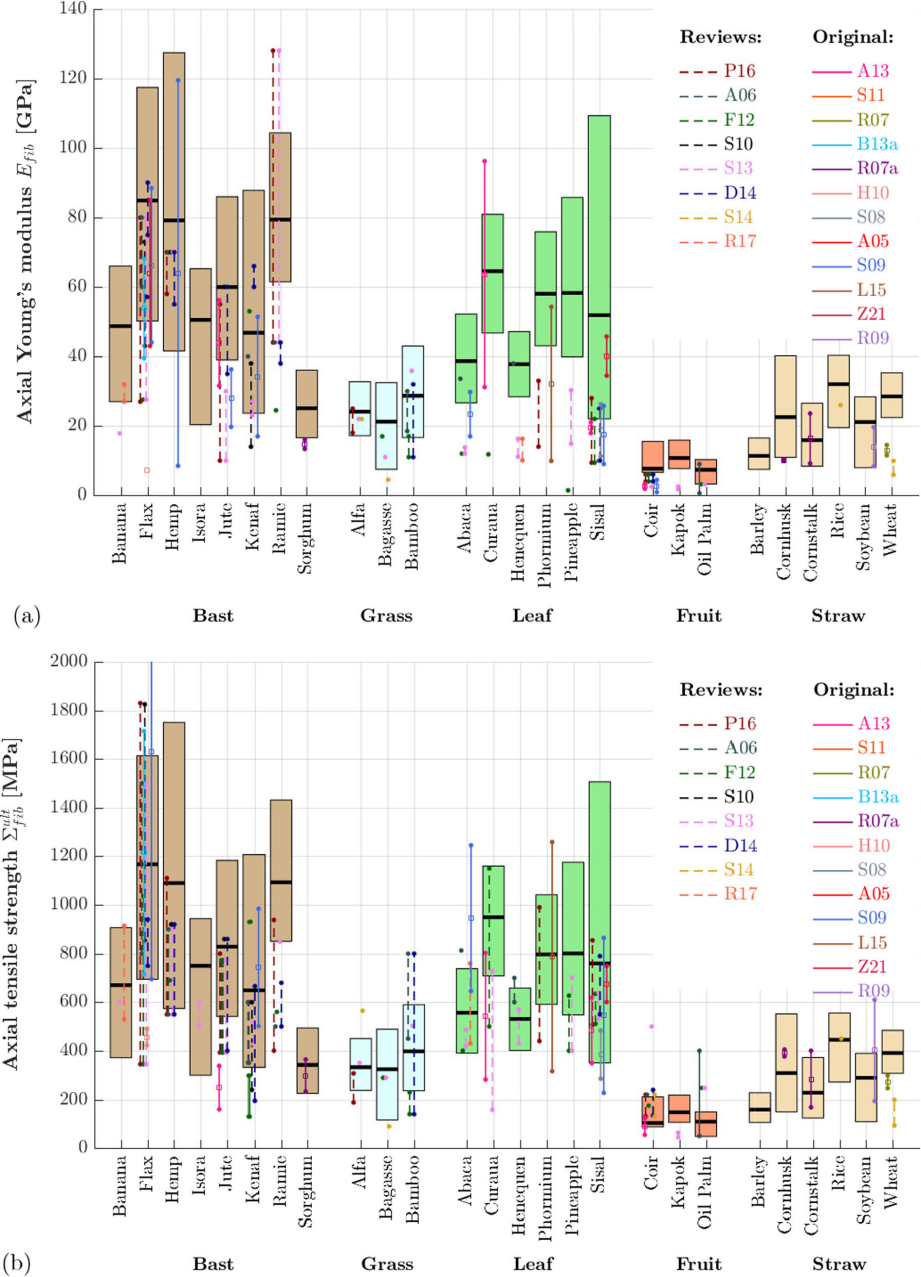
Model validation for **a** axial Young’s modulus and **b** axial tensile strength: comparison of model predictions (colored bars represent predicted intervals between the minima and maxima modulus/elastic limit, black horizontal lines represent representative averages, see Tables 2-4 for the corresponding minimum/average/maximum physicochemical fiber properties) with experimental data gathered from published reviews (P16...[5], A06...[93], F12...[2], S10...[120], S13...[12], D14...[4], S14...[98], R17...[9]; dashed lines) and from original tests (A13...[10], S11...[11], R07...[71], B13a...[117], R07a...[99], H10...[61], S08...[118], A05...[116], S09...[13], L15...[106], Z21...[119]; continuous lines)

In order to validate the model-predicted mechanical fiber properties, we first report on published experimental results, obtained from single fiber testing. Single fiber testing is a challenge as such, experimental difficulties arise (i) from fiber slippage or imperfect fiber alignment, see e.g. [13, 115] for more discussion, (ii) from simplification regarding the quantification of the cross section area of the fibers [10, 61], or (iii) from size effects related to gauge lengths [116], which may be removed when accounting for the machine compliance [10]. Given the experimental challenges, reported mechanical properties vary significantly, even more so than the physicochemical properties discussed in Sec. 2.4. In order to cope with this variety, we herein concatenate experimental data from several sources, including original test data [10, 11, 13, 61, 71, 85, 99, 106, 116–119] and data previously collected in review papers [2, 4, 5, 9, 12, 93, 98, 120]. Mechanical properties for 25 out of the 26 fibers have been found, data for barley straw fibers is not available. The reported ranges of mechanical properties are depicted in Fig. 4, whereby solid lines refer to original data and dashed lines to data from reviews.

Model-predicted Young’s moduli $E_\text {fib}$ and tensile elastic limits ${\Sigma }_\text {fib}^\text {ult}$, respectively, are obtained from stiffness homogenization according to Eqs. (8–13) and strength homogenization according to Eqs. (14–15), evaluated for plant-specific physicochemical properties given in Tables 2, 3 and 4, and for intrinsic (plant-independent) mechanical phase properties (stiffness according to Table 1, cellulose nanofibril strength according to Sec. 2.3). In Fig. 4, the predicted properties are depicted by colored bars, representing predicted intervals between the minimum and maximum, with black horizontal lines, representing the predicted property from average physicochemical input properties, see Tables 2, 3 and 4 for the corresponding minimum/average/maximum physicochemical fiber properties. Model-predicted ranges for both the elastic modulus and the elastic limit are generally very close to the experimentally measured ranges for elastic modulus and tensile strength, respectively. In more detail, the model is able to reproduce the extraordinarily high mechanical properties seen in most bast fibers, as well as the rather low properties of grass, fruit, and straw fibers. This does corroborate that intrinsic mechanical phase properties gathered from molecular simulations and nanoscale testing can be successfully translated to macroscopic fiber properties—if microstructural features of plant fibers are suitably represented, as done so by the developed multiscale model shown in Fig. 2.

Not all experimentally measured fiber properties fall within the predicted ranges. While predicted and measured strength ranges are generally very close, predicted moduli are typically slightly larger than the experimentally measured ones. This might be explained, on the one hand, by the aforementioned difficulties related to single fiber testing such as fiber slippage, which are likely to affect the stiffness test results more than the strength results, and moreover, always lead to experimental values below the actual elastic fiber modulus [13]. The stiffness overestimation might, on the other hand, be caused by assigning the molecular dynamics-derived stiffness of perfectly regular crystalline cellulose to the nanofibril phase, despite the interfaces/defects present in nanofibrils [14]. Moreover, some bio-physicochemical features such as sugar contents are not considered in the model, but might partly explain e.g. the smaller experimentally determined moduli and strengths for hemp compared to flax [62, 121]. Modeling the cell wall layers explicitly rather than considering a homogeneous phase, as e.g. done in [24] might also lead to a better performance, but is limited by quantitative experimental data on layer-specific physicochemical properties.

**Fig. 5:**
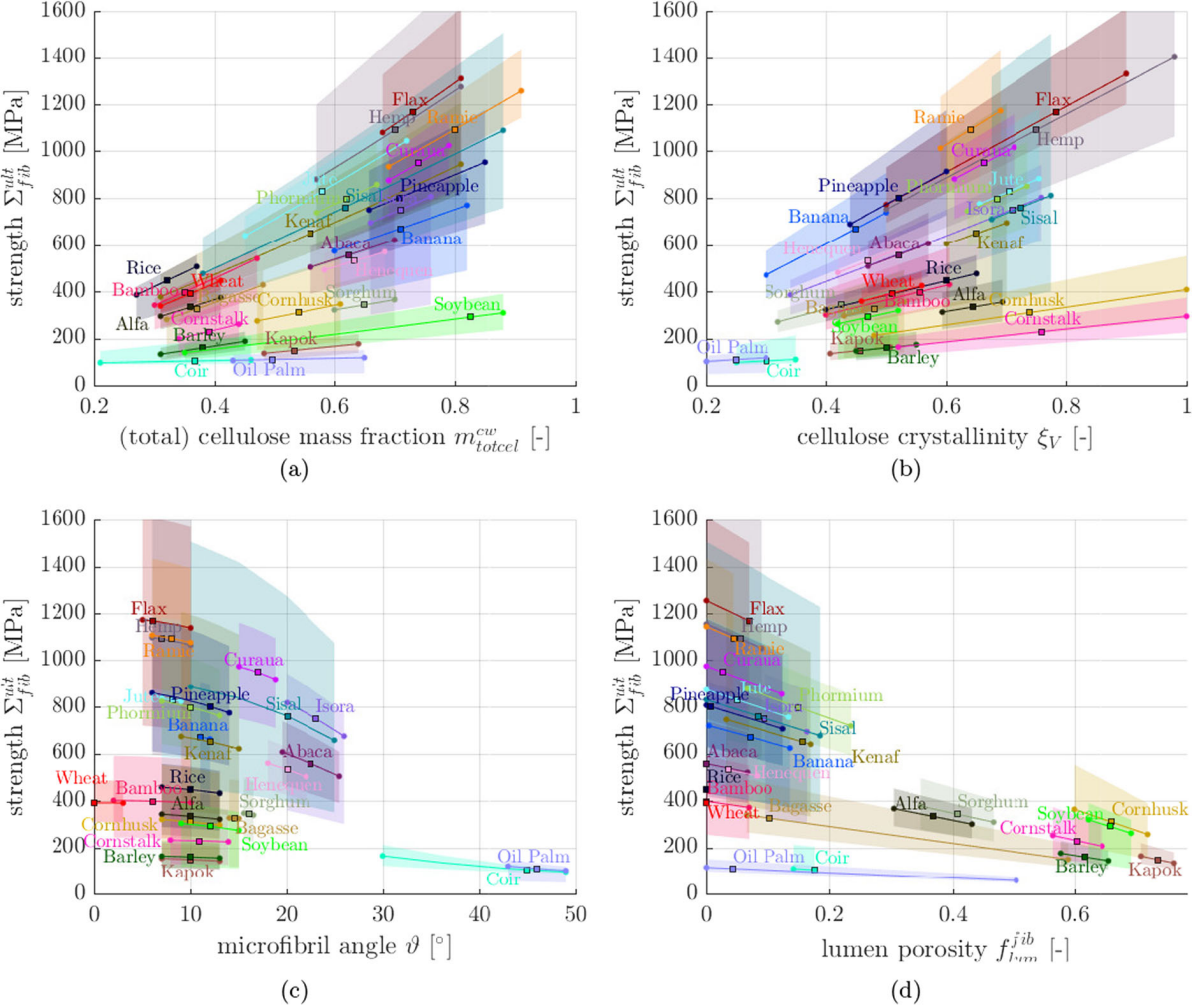
Sensitivity of model-predicted fiber strengths with respect to changes of the **a** cellulose mass fraction, **b** cellulose crystallinity, **c** microfibril angle, **d** lumen porosity; colored solid lines refer to average physicochemical properties, colored areas span the intervals between minimum and maximum properties, and square points represent the prediction for average properties

By providing a quantitative link between microstructural features and macroscopic fiber properties, the model further allows us to understand and explore the origin of the observed stiffness and strength differences between the different fibers, as discussed next. Most bast fibers, particularly flax, hemp, and ramie exhibit an outstanding mechanical performance, with predicted average moduli amounting to approximately 90 GPa and predicted average strengths amounting to approximately 1100 MPa. Their microstructure, characterized by high cellulose contents [see Table 2 and the sensitivity diagram in Fig. 5a], high cellulose crystallinities [Table 3 and Fig. 5b], small microfibril angles [Table 3 and Fig. 5c], and small lumen porosities resulting in high fiber densities [Table 3 and Fig. 5d], is tailored to maximize their mechanical performance in fiber direction. The low modulus and strength of sorghum bast fibers, in turn, result mainly from the high lumen porosity. Grass, fruit, and straw fibers exhibit rather low mechanical properties, see Fig. 4. The predicted properties of coir and oil palm fibers are the lowest among the 26 studied fibers, with (average) moduli below 8 GPa and (average) strengths close to 100 MPa, which nicely matches the available experimental data. They both suffer from the highest microfibril angles found in all plant fibers, with averages amounting to roughly 40-45$^\circ$ [see Fig. 5c], coupled with small crystalline cellulose volume fractions [Table 4 and Figs. 5a,b]. Kapok fibers, as well as straw fibers from barley, corn, and soybean exhibit lumen porosities above 60 % (Table 3), which prevents the good mechanical performance of their cell walls to translate to the fiber scale.

Finally, the sensitivity of the fiber strength^1^ with respect to changes of the fibers’ physicochemical properties is discussed. The dependencies are studied for four significant input properties: (a) the cell wall-related mass fraction of (crystalline + amorphous = total) cellulose $m_\text {totcel}^{cw}$, (b) the volumetric cellulose crystallinity $\xi _V$, (c) the microfibril angle $\vartheta$, and (d) the lumen porosity $f_\text {lum}^\text {fib}$. In more detail, we consider that one of the four properties exhibits values bound by the interval between minimum and maximum values reported in the literature (see Tables 2 and 3). Considering that all other physicochemical properties exhibit average input values, we arrive at the solid lines in the fiber-specific influence diagrams shown in Fig. 5. Considering, in turn, that all other input properties still maintain their variability within the corresponding intervals, we obtain the fiber-specific colored areas of Fig. 5. The strengths of all 26 fibers monotonously increase with increasing cellulose mass fractions, with increasing crystallinity, with decreasing microfibril angle, and with decreasing lumen porosity. Moreover, Fig. 5 reveals the origin of the variability of the predicted strength results. A significant share of the variability results from the broad intervals of the cellulose mass fractions, which originate e.g. from different fiber extraction methods. This shows that increasing the cellulose content of fibers, e.g. by means of chemical treatments to remove wax, hemicellulose, and/or lignin, is a very effective way of enhancing the fiber strength, as corroborated by single fiber tests [122, 123]. The microfibril angle, in turn, even though its actual quantity has a significant importance, typically ranges within narrow intervals (except for Sisal), such that the variability of the microfibril angle has little effect on the strength variability.

## Conclusions and outlook

An established multiscale modeling framework for natural fibers based on continuum micromechanics [25, 27, 30] is herein adopted to predict the axial mechanical properties of 26 of the most commonly used plant fibers. Relying on a plant-independent microstructural representation but plant-specific physicochemical fiber properties, which even for a given plant species may vary considerably, nanoscale mechanical cellulose properties (170 GPa axial modulus, 2300 MPa axial strength) are upscaled to the macroscopic fiber scale. In more detail, we predict upper and lower bounds of the axial mechanical fiber properties, based on reported intervals of physicochemical input properties. Predicted axial mechanical properties amount to moduli below 10 GPa and strengths below 100 MP for fibers with large microfibril angles, high lumen porosities, and/or low (crystalline) cellulose contents, as found in fibers from fruits, seeds, and straws. However, predicted moduli can be as high as 120 GPa and predicted strengths can be as high as 1600 MP for bast fibers with ideal physicochemical properties regarding stiffness and strength in the longitudinal direction. The predicted bounds, for almost all 26 studied plant fibers, frame the experimentally determined fiber stiffnesses and strengths, respectively, which were gathered from published single fiber test campaigns. This way, we corroborate that both the reduction of the mechanical performance upon transition from the nanoscale to the macroscale as well as the differences in mechanical properties among the fibers from different plants can be assessed quantitatively when incorporating the main microstructural features such as microfibril angle, cellulose crystallinity, and lumen porosity.

Future work aims at expanding this micromechanics model to biocomposites by including yet another macroscopic scale of observation. At this scale, plant fibers of any orientation are interacting with the surrounding matrix phase, whereby modeling of imperfect bonding at fiber-matrix interfaces might be incorporated. In this sense, the proposed model for plant fibers is intended as a contribution to the three-dimensional mechanical description of biocomposites, which may pave the way to new and improved composite formulations. A reliable description of the mechanical composite behavior is particularly important for developing and optimizing lightweight construction elements from such materials. Moreover, emphasis should be also put on incoporating fracture mechanics and stochastics into the description of the failure process of cellulose-based fibers, supported by novel experimental characterization attempts of cellulose nanofibrils.

## Appendix A: hill tensors

We deal with Eshelby problems involving spheroidal inclusions *i* in matrices with stiffness tensor $\mathbb {C}{}_m$. Associated Hill tensors $\mathbb {P}{}_i^m$, required for micromechanics homogenization and concentration relations according to Eqs. (8–13), read as

A1 $$\begin{aligned} \mathbb {P}{}_i^m=\mathbb {S}{}_i^m:\mathbb {C}{}_{\rm {m}}^{-1}\,, \end{aligned}$$

whereby $\mathbb {S}{}_i^m$ denotes the Eshelby tensor and is a function of the inclusion’s shape (cylindrical $i=\text {cyl}$, or spherical $i=\text {sph}$) and of the matrix’ Poisson’s ratio $\nu _m$ [52]. As for spherical inclusions embedded in a polymer network matrix, the Eshelby tensor components read as [32]

A2 $$\begin{aligned} \begin{aligned} S_{\text {sph},ijkl}^\text {pm}=&\frac{5\nu _\text {pm} - 1}{15\left( 1-\nu _\text {pm}\right) }\delta _{ij}\,\delta _{kl} + \\&\frac{4 - 5\nu _\text {pm}}{15\left( 1-\nu _\text {pm}\right) }\left( \delta _{ik}\,\delta _{jl} + \delta _{il}\,\delta _{jk}\right) \,, \end{aligned} \end{aligned}$$

with $\delta _{ij}$ denoting the Kronecker delta. As for the cylindrical cellulose inclusions (nanofibrils and microfibrils, respectively), the infinite matrix in the corresponding Eshelby problem is isotropic, such that non-zero components with respect to the local orthonormal coordinate base $x_1,x_2,x_3$ (with $x_3$ as the cylinder axis direction) read as [124]

A3 $$\begin{aligned} S_{{{\text{cyl}},2222}}^{m} & = S_{{{\text{cyl}},3333}}^{m} = \frac{{5 - 4\nu _{m} }}{{8{\mkern 1mu} \left( {1 - \nu _{{\text{m}}} } \right)}} \\ S_{{{\text{cyl}},2233}}^{m} & = S_{{{\text{cyl}},3322}}^{m} = \frac{{ - 1 + 4\nu _{m} }}{{8{\mkern 1mu} \left( {1 - \nu _{{\text{m}}} } \right)}} \\ S_{{{\text{cyl}},1313}}^{m} & = S_{{{\text{cyl}},1212}}^{m} = \frac{1}{4} \\ S_{{{\text{cyl}},2323}}^{m} & = \frac{{3 - 4\nu _{m} }}{{8{\mkern 1mu} \left( {1 - \nu _{{\text{m}}} } \right)}}{\mkern 1mu} , \\ \end{aligned}$$

whereby m stands either for the amorphous cellulose matrix (for nanofibril inclusions), or for the polymer network matrix (for microfibril inclusions). As for the cylindrical lumen pores, the infinite matrix in the corresponding Eshelby problem is transversally isotropic, whereby the cylinder axis is aligned with the matrix’ axis of transverse symmetry. This way, the the Eshelby tensor reads as [125]

A4 $$\begin{aligned} \begin{aligned} \mathbb {S}{}_\text {cyl}^\text {cw}=&\frac{C_{\text {cw},1}}{2C_{\text {cw},1}+C_{\text {cw},2}}\mathbb {T}{}^{(1)} +\frac{C_{\text {cw},1}+C_{\text {cw},2}}{2C_{\text {cw},1}+C_{{\text {cw}},2}}\mathbb {T}{}^{(2)}\\&+\frac{C_{\text {cw},3}}{2C_{\text {cw},1}+C_{\text {cw},2}}\mathbb {T}{}^{(3)} + \mathbb {T}{}^{(5)}\,. \end{aligned} \end{aligned}$$

With $x_3$ as the axis of transverse symmetry, tensors $T^{(1)},\dots ,T^{(5)}$ have the following non-zero components

A5 $$\begin{aligned} \begin{aligned} T_{1111}^{(1)}&=T_{2222}^{(1)}=T_{1122}^{(1)}=T_{2211}^{(1)}=1\\ T_{1212}^{(2)}&=T_{2121}^{(2)}=T_{1221}^{(2)}=T_{2112}^{(2)}=T_{1111}^{(2)}=T_{2222}^{(2)}=\\&-T_{1122}^{(2)} =-T_{2211}^{(2)}=\frac{1}{2}\\ T_{1133}^{(3)}&=T_{2233}^{(3)}=1\\ T_{1313}^{(5)}&=T_{2323}^{(5)}=T_{1331}^{(5)} =T_{2332}^{(5)}=T_{3113}^{(5)}=T_{3223}^{(5)}=\\&T_{3131}^{(5)}=T_{3132}^{(5)}=\frac{1}{4}\,. \end{aligned} \end{aligned}$$

and $C_{\text {cw},1}$, $C_{\text {cw},2}$, and $C_{\text {cw},3}$ read as

A6 $$\begin{aligned} \begin{aligned} C_{\text {cw},1}&=\left( C_{\text {cw},1111}+C_{\text {cw},1122}\right) /2\,,\\ C_{\text {cw},2}&=2\,C_{\text {cw},1212}\,,\\ C_{\text {cw},3}&=C_{\text {cw},1133}\,, \end{aligned} \end{aligned}$$

## Footnotes

1 The sensitivity of the fiber stiffness is very similar to the one of the strength and is therefore not discussed in detail
